# The Book World of Medicine and Science

**Published:** 1893-10-07

**Authors:** 


### The Book World of Medicine and Science.

Crime and its Causes. By William Douglas Morrison, of H.M. Prison, Wandsworth. (London: Swan Sonnenschein and Co., Paternoster-square.)

The criminal cannot complain of being neglected by his betters in this age. He is the object of the most careful, scientific, and sympathetic observation, a large proportion of which is intended to prove that his moral obliquity is really a physical misfortune, and that if punishment be required, we ought in justice to adopt the converse of that Chinese practice which ennobles the ancestors of the virtuous man, and punish, if possible, the ancestors of the vicious. Mr. Morrison, who writes from the vantage ground of fourteen years' practical acquaintance with prisoners, speaks less of dolicocephalic heads and more of innate evil tendencies than Professor Lombroso, but both agree in regarding crime as a problem which all our philanthropic efforts have hitherto failed to solve. Crime is increasing as rapidly as population, in some countries indeed more rapidly, and while climate and race have a certain modifying power, it does not seem that advancing civilisation is inevitably accompanied by advancing morality. Statistics cannot be regarded as infallible guides on this subject; for, as Mr. Morrison points out, similar crimes are not held in the same estimation in all countries, nor even in all parts of the same kingdom, and are therefore not hunted down with equal energy or punished with equal severity. But judging from such a crime as murder, on which the opinion of the civilised world is tolerably unanimous, it does not appear that there is any diminution in frequency proportionate to the diminution of temptation. The same holds good of other crimes, in so far as statistics on these can be collated and trusted. Socialists, and gentle souls of all political and religious creeds are fond of arguing that poverty makes criminals; that a man steals only because he is hungry; strikes a blow only because he feels that society has wronged him. But Mr. Morrison shows very clearly that destitution is not the invariable or even general excuse for crime. Crime and pauperism reach their highest point in the summer and autumn months, when living is easiest and work most easily obtained by even the poorest and most untrained of the community. In fact, the comparative abundance of work may almost be taken as tending to increase criminal propensities. Work means money, and money, in the lowest classes, means drink, and drink means crime. Nor by crime do we mean simple imprisonment for drunkeness, because the same causes which make drink attainable enable the drunkard to escape incarceration by paying a fine. But his intoxication too often leads him to commit those crimes which fines do not expiate.

Mr. Morrison makes it, indeed, abundantly clear that it is not poverty that makes the criminal. The working man in distress, reduced against his will to beg, and usually accompanied by six children, all, except the inevitable baby in arms, apparently about four years of age, is a fraud and a myth. "It is no more the practice of working men to go about begging than it is the practice of the middle class," says Mr. Morrison, "but until this elementary fact can be laid hold of by the public all statutory enactments for the suppression of mendicity will be but partial in their operation." He gives a powerful illustration of this from some

THE BOOK WORLD OF MEDICINE AND SCIENCE—*continued.*

statistics collected by M. Monod, of the Ministry of the Interior of France. A benevolent French citizen wished to give the chance they professed to desire to any able-bodied vagrants who uttered the old cry of "Can't get work to do." He made arrangements with various merchants and manufacturers to take on any man who came with a letter of recommendation from him, and to pay them (doubtless out of his pocket) the respectable wage of four francs a day. In eight months he dealt with 727 cases. Each of these beggars was told to come back the next day for the necessary letter of recommendation. Of the total number more than half (415) never came back for the letter; 138 came back for it, but never presented it. Of the remainder, some worked half a day, demanded their two francs, and left; a few worked the whole day and then disappeared. Of the whole 727, only 18 were found at work at the end of the third day. So much for the out-of-work man, who is, in the vast majority of cases, neither more nor less than a sturdy beggar.

It is evident that Mr. Morrison does not look upon the average criminal as a fallen angel or an injured innocent, and he admits that for the hardened offender all that society can do is to seclude them in such a fashion that they shall not prey upon their neighbours. But he is no mere pessimist, and in many cases, especially of first conviction, he thinks that good work is being done by Prisoners' Aid Societies, which send the discharged criminal to his home, redeem pawned tools for him, try to obtain work for him, and, when it seems judicious, help him to emigrate. Unfortunately, these societies are not popular charities, and their operations are limited by insufficiency of funds. That a practical man who does not take too roseate a view of the subject, like the author of this interesting and thoughtful book, regards them as worthy of support, should recommend these Aid Societies to the charitable public.

Practical Kindergaten Lessons for English Infant Schools. By Mrs. E. Mortimer. (J. Hughes and Co., Pilgrim Street, Ludgate Hill.)

In Mrs. Mortimer's capital little book on kindergarten lessons, we have before us something practicable and tangible. The aim of this work is to induce some of our numerous private schools, which play a far greater part in the vast machinery of education than is generally realised, to induce these to lift a hand in the work of training little ones from very infancy to be bright, observant, practical units of the world around them. Mrs. Mortimer has laid aside the usual technical language of "system-advocates," and explains to us in plain English the elements of the cause she upholds, trying at the same time to fire her readers with the true "Fröbelian spirit," and showing herself a worthy disciple of one who gave his life to [the establishment of a "children's garden," in which he and his followers might live *for* and *with* the little ones of generations to come. The writer goes on to explain simply and clearly the object of introducing the system, and urges that, even if the entire course cannot be followed in many cases, at least the *spirit* of the practical teaching may be worked into all the lessons. Young teachers in particular would profit much by the hints given in this little book, for they enable them to fix the attention of their pupils by exciting the interest and sympathies of the children; whilst to any who wish to adopt the Fröbel method of training, Mrs. Mortimer's advice will be most welcome and her instructions clear, concise, and helpful, since she meets and, to a great extent, smooths away the difficulties which present themselves to anxious heads of the smaller schools.

